# De Witt County Medical Society

**Published:** 1858-02

**Authors:** 


### PROCEEDINGS OF MEDICAL SOCIETIES.

### DE WITT COUNTY MEDICAL SOCIETY.

The society met in quarterly session at the schoolhouse in Marion, January 4th, 1858, at 2 o'clock p. m.

Members present—Drs. J. W. Redden, T. K. Edmiston, J. B. Hunt, J. C. Ross, John Wright, B. S. Lewis, E. Richards, J. H. Tyler, Z. H. Madden, W. W. Adams, J. McHugh, J. J. Lake, and Christopher Goodbrake.

The President, Dr. Adams, in the chair.

The minutes of the previous meeting were read and approved.

Dr. Lake reported a very interesting case of puerperal disease, which was discussed by Drs. Edmiston, Tyler, Lake and Goodbrake.

Dr. Ross reported a remarkable case of retained placenta. It seems that three sacks or pouches—in shape and size resembling the finger of a medium-sized glove — had formed in the uterus, into which portions of the placenta had intruded, and become strangulated, by the mouths of the pouches being constricted, and firmly holding the portions of the placenta within them. In addition to this, a large portion of the placenta was adherent to the fundus uteri so firmly that it was impossible to remove the whole of it with the hand, without using too much force. Ergot was also administered without any good result. The case was left to nature, with the exception of injections, to correct the offensive discharge, and medicine to allay a slight irritative fever which ensued. The woman got along well.

Dr. Lewis read a valuable essay on the "*Pathology and Treatment of Phthisis Pulmonalis.*" The essayist had evidently studied the disease well. He gave a *resume* of the different theories advanced in regard to the pathology of this insidious destroyer of mankind, and also of the treatment which has from time to time become fashionable. He deprecated the idea of other diseases terminating in phthisis, where there was not a "previous tendency" to tuberculosis. The Doctor thinks that iodine may be of service to correct the tubercular diathesis, but is certain that it does harm after the tubercles have formed, and more especially when they begin to soften. He recommends, as the only available treatment, where the disease has once fairly set in, tonics, with plenty of fresh air, exercise, and nourishing diet.

The essay was discussed by several of the gentlemen present.

Dr. Redden read an essay on "*Man Physically and Intellectually Considered.*" The essay was well written, and was listened to with much interest.

Dr. Ross read an essay on the "*Pathology and Treatment of Typhoid Fever.*" The essayist advocated the abortive treatment at the commencement of the disease. He believes that the disease can, in almost all cases, be "broken up" by large doses of quinine. If the disease should continue in spite of this treatment, then to continue the quinine in smaller doses throughout the disease, in connection with turpentine, etc. The Doctor says he is very certain that mercury, in any form, is never administered with any benefit whatever in this disease.

The essay called forth an animated discussion, in which most of the gentlemen present partook, pending which the society adjourned until half-past 6 o'clock.

EVENING SESSION.

The President called the society to order at half-past 6 o'clock. The discussion on the essay of Dr. Ross was continued by most of the members present.

Dr. Edmiston did not believe that typhoid fever could be cut short. He thought the disease specific in its character, and that Peyer's glands became affected from the outset; that the lesion of the mucous follicles and glands was, in fact, *the* disease, and must run a certain course; and all that the physician could do was to husband the strength of the patient, and treat the symptoms as they presented themselves. He never could derive any benefit from quinine, except in the last stages of the disease, and thought calomel, hydrarg. cum cretia, and mass. pil. hydrar. (whichever was most indicated) could be administered with great benefit—if proper *discrimination* was not lost sight of—throughout the different stages of the disease under consideration.

Dr. Goodbrake thought it not impossible to cut short an attack of fever. He did not believe that Peyer's glands necessarily became involved in all cases of this disease. He thought, because a patient had typhoid fever, it did not always follow that he had to be sick a certain number of days or weeks; but had seen patients laboring under well established typhoid fever get well at all intermediate spaces of time, in from a few days to several weeks. He did not believe in treating diseases by their names, but endeavored to treat symptoms as they appeared throughout any disease; and he believed the pathological conditions, as evidenced by the symptoms, usually observable in typhoid fever contradicted the administration of mercurials. He believed the views set forth in the essay mainly correct.

Dr. McHugh held, that in the disease under consideration, there was, in a certain degree, from the very first, great nervous prostration, an impoverished state of the blood, and a want of proper tonicity in all the assimilative and excretive organs; and, consequently, we should make choice of such remedies, and make use of such means as would brace up the nervous system, and enrich the blood. And, as a matter of course, calomel—judging from its well known effects upon the blood, when given so as to produce its specific action upon the system, could have no good, but, on the contrary, have a bad effect.

Drs. Adams and Hunt agreed in a great degree with the essayist.

Drs. Richards, Lewis and Madden held views similar to those expressed by Dr. Edmiston.

On motion of Dr. Edmiston, the thanks of the society were tendered to Drs. Ross and Lake for their reports, and to Drs. Lewis, Redden and Ross for their essays.

Dr. Richards moved that when this society adjourns, it shall be to meet on the first Monday in May, instead of the first Monday in April. Carried.

On motion of Dr. Madden, the society went into an election for delegates to the American Medical Association, when Joseph C. Ross, M. D., and Christopher Goodbrake, M. D., were elected, and each one authorized to appoint a substitute in case he cannot attend.

On motion of Dr. Richards, the following resolution was adopted:

*Resolved*, That at each meeting of this society the President shall announce a subject for discussion at the next meeting of this society.

In conformity with the above resolution, the President announced *Stomatitis Materni* as the subject for discussion at the next meeting of the society.

The President appointed Drs. Wright and Richards essayists at the next meeting.

On motion of Dr. Redden, the thanks of the society were tendered to Drs. Lewis and Lake, and, through them, to their ladies, for the very liberal hospitality extended to the members of this society during the present session.

On motion, a copy of these proceedings was ordered to be furnished the *Chicago Medical Journal* for publication.

On motion, the society adjourned, to meet in annual session, at Clinton, on the first Monday of May next, at 10 o'clock a.m.

